# Editorial: Interdisciplinary approaches to address mental health concerns during menopause

**DOI:** 10.3389/frph.2026.1921443

**Published:** 2026-07-21

**Authors:** Shagaf H. Bakour, Lisa Taylor-Swanson

**Affiliations:** 1 Aston Medical School, College of Health and Life Sciences, Aston University, Birmingham, United Kingdom; 2 The Institute of Clinical Sciences, University of Birmingham, Birmingham, United Kingdom; 3 Department of Obstetrics and Gynaecology, Sandwell and West Birmingham Hospitals NHS Trust, Birmingham, United Kingdom; 4 College of Nursing, University of Utah, Salt Lake City, UT, United States

**Keywords:** bone health, interdisciplinary, memory, menopause, mental health

Menopause is a universal life transition, yet its psychological and emotional dimensions remain too often under-recognised in clinical practice, research, education and public discourse. For many women, menopause is not experienced simply as a collection of physical symptoms, but as a complex period in which bodily change, mood, sleep, cognition, confidence, identity, relationships and wider life pressures may all intersect.

When I was invited by the journal Editorial Board to lead this Research Topic, my motivation came from more than the expanding scientific literature. It was also rooted in my own clinical experience. In practice, I have repeatedly seen women present with menopausal symptoms where the physical, emotional and psychological dimensions were deeply connected. These encounters reinforced for me that menopause is not only a biological transition, but also a period in which mental health, identity, wellbeing and quality of life can be profoundly affected. Anxiety, low mood, sleep disturbance, pain, loss of confidence and emotional vulnerability were often not separate concerns, but part of the same lived experience. These encounters made clear to me that menopause and mental health should not be viewed in isolation. This was the reason I wanted to shine a light on this association through a dedicated Research Topic.

This collection, co-edited with Lisa Taylor-Swanson, brings together six thoughtful contributions: four fully published articles and two recently accepted articles. Together, they provide a rich and timely exploration of mental health concerns during menopause from clinical, psychological, social, educational and policy perspectives. Importantly, the collection has now met the required threshold for an Editorial, allowing us to reflect on the emerging messages across the Topic as a whole.

The Research Topic opens with important epidemiological evidence on anxiety and depression among menopausal women. In *A study on anxiety and depression symptoms among menopausal women: a web-based cross-sectional survey*, Kandasamy et al. report substantial levels of anxiety and depressive symptoms among menopausal women in the Asir region of Saudi Arabia. Their work highlights the need for better awareness, screening and culturally sensitive support, particularly in settings where stigma, limited health literacy or lack of access to services may prevent women from seeking help.

Gao et al. extend this discussion in *Depression symptoms in perimenopausal women with somatic pain: nomogram construction based on a logistic regression model*. Their study focuses on perimenopausal women with somatic pain and identifies factors associated with depressive symptoms, including pain distress, sleep deprivation, self-perceived health, life satisfaction and daily functioning. This article is especially valuable because it reflects a common clinical reality: emotional distress during menopause is often expressed through, and intensified by, physical symptoms. It reminds clinicians that pain, sleep, mood and function should be assessed together rather than treated as unrelated complaints.

The physical and psychological dimensions of menopause are also brought together in *Impact of menopause hormone therapy, exercise, and their combination on bone mineral density and mental wellbeing in menopausal women: a scoping review*. Platt et al. explore the evidence around menopause hormone therapy, exercise and their combined effects on bone mineral density and mental wellbeing. This contribution reinforces the importance of integrated care and balanced counselling. Menopause care should not focus narrowly on symptom control alone, but should support long-term physical health, emotional wellbeing and quality of life.

The collection also broadens the conversation by considering experiences that are frequently overlooked. In *Experiences of autistic women in menopause: brief review and recommendations for practice and research*, Badgett et al. examine the experiences of autistic women during menopause. Their work draws attention to sensory sensitivities, interoceptive differences, communication needs and the lack of autism-informed menopause care. This article is an important reminder that inclusive menopause support requires clinicians to listen carefully, adapt communication and recognise that women may experience and describe menopausal symptoms in very different ways.

Two recently accepted contributions add further depth to the Research Topic and should be recognised as integral to the collection. Sánchez-Ortega et al., in *Exploring Women's Experiences and Needs During the Climacteric to Enhance Positive Mental Health and Self-Care*, centre women's lived experiences during the climacteric. Their work highlights the need for clear information, self-care support, validation and holistic approaches that promote positive mental health. This perspective is particularly important because mental wellbeing is not simply the absence of clinical anxiety or depression. It also encompasses confidence, self-understanding, voice, the ability to make informed choices and the capacity to navigate change with appropriate support.

Finally, Etsey et al., in *American Medical Women's Association's Commitment to Advancing Menopausal Health Equity and Awareness*, place menopause within a wider agenda of health equity and public awareness. Their perspective draws attention to persistent gaps in clinician education, access to care, insurance coverage, workplace recognition and culturally competent support. This article makes clear that improving menopause-related mental health is not only a matter of individual clinical practice; it also requires system-level change.

Across the six contributions, several shared messages emerge. First, mental health assessment should be a routine and normal part of menopause care. Second, symptoms such as sleep disturbance, pain, vasomotor symptoms, cognitive concerns, mood changes and sexual wellbeing should be considered together. Third, interdisciplinary care is essential, drawing on gynaecology, primary care, psychiatry, psychology, nursing, physiotherapy, public health and community education. Fourth, menopause research and services must become more inclusive, particularly for women whose experiences are shaped by neurodiversity, culture, socioeconomic disadvantage or barriers to care.

This Research Topic does not present menopause as a disorder. Rather, it recognises menopause as an important developmental life transition that can affect mental health, intensify existing vulnerabilities and create opportunities for prevention, education and empowerment. The articles in this collection collectively call for more compassionate conversations, earlier recognition, better-informed clinicians and more equitable systems of care.

Women should not have to separate their physical, emotional and social experiences before receiving help. Menopause care must be designed around the whole person. If this Research Topic helps to move practice, research and policy in that direction, then it will have fulfilled its purpose.

